# Sex difference in dopamine D1-D2 receptor complex expression and signaling affects depression- and anxiety-like behaviors

**DOI:** 10.1186/s13293-020-00285-9

**Published:** 2020-02-22

**Authors:** Ahmed Hasbi, Tuan Nguyen, Haneen Rahal, Joshua D. Manduca, Sharon Miksys, Rachel F. Tyndale, Bertha K. Madras, Melissa L. Perreault, Susan R. George

**Affiliations:** 1 Department of Pharmacology, University of Toronto, Toronto, ON Canada; 2 Department of Medicine, University of Toronto, Toronto, ON Canada; 3 Molecular and Cellular Biology, University of Guelph, Guelph, ON Canada; 4 Campbell Family Mental Health Research Institute, Centre for Addiction and Mental Health, Toronto, ON Canada; 5 Department of Psychiatry, University of Toronto, Toronto, ON Canada; 6 Department of Psychiatry, Harvard Medical School, Boston, MA USA; 7 McLean Hospital, Belmont, USA

## Abstract

Depression and anxiety are more common among females than males and represent a leading cause of disease-related disability in women. Since the dopamine D1-D2 heteromer is involved in depression- and anxiety-like behavior, the possibility that the receptor complex may have a role in mediating sex differences in such behaviors and related biochemical signaling was explored.

In non-human primate caudate nucleus and in rat striatum, females expressed higher density of D1-D2 heteromer complexes and a greater number of D1-D2 expressing neurons compared to males. In rat, the sex difference in D1-D2 expression levels occurred even though D1 receptor expression was lower in female than in male with no difference in D2 receptor expression. In behavioral tests, female rats showed faster latency to depressive-like behavior and a greater susceptibility to the pro-depressive and anxiogenic-like effects of D1-D2 heteromer activation by low doses of SKF 83959, all of which were ameliorated by the selective heteromer disrupting peptide, TAT-D1. The sex difference observed in the anxiety test correlated with differences in low-frequency delta and theta oscillations in the nucleus accumbens. Analysis of signaling pathways revealed that the sex difference in D1-D2 heteromer expression led to differences in basal and heteromer-stimulated activities of two important signaling pathways, BDNF/TrkB and Akt/GSK3/β-catenin.

These results suggest that the higher D1-D2 heteromer expression in female may significantly increase predisposition to depressive-like and anxiety-like behavior in female animals.

## Introduction

The World Health Organization [1] estimated that globally, the total number of people with depression exceeded 300 million in 2015, representing ~ 4.4% of the global population. A similar proportion also suffers from anxiety disorders but many people experience both conditions simultaneously indicating substantial comorbidity. Depressive disorders are the single largest contributor to disease-related disabilities and the major contributor to suicides [1]. Globally, depression is more common among females (5.1%) than males (3.6%) [1] and the leading cause of disease-related disability in women (reviewed, [2, 3]). A consistent finding in psychiatric epidemiology shows women have higher rates of major depression and are twice as likely as men to develop it. Women have a lifetime prevalence for major depressive disorder reaching 21.3%, compared to 12.7% in men [3]. Anxiety disorders are more common among females than males (4.6% compared to 2.6% worldwide) according to the WHO [1].

The sex difference starts around puberty (reviewed in [3–5]), prominent between ages 15 and 18 [5, 6]. The mechanisms leading to the higher prevalence of depression and anxiety in women are not completely understood. However, the female predisposition to depression is thought to involve different biological processes, such as genetic vulnerability, hormonal fluctuations linked to reproductive function, and psychosocial parameters, e.g., social status, role-stress, victimization, and coping style, to cite a few [3, 5, 7–10]. There are sex differences also in the presentation, course of illness as well as drug sensitivity and treatment responses to medication and psychotherapy [3, 9, 10]. The core clinical features of disorders such as depression in humans are subjective experiences, rather than observable behaviors. Nevertheless, several animal models have been developed to test depression-like behavior, such as by inducing learned helplessness, maternal separation, chronic restraint, and chronic unpredictable stress; these models are also utilized to induce anxiety-like behavior [9]. Although sex differences in animal model systems and humans were shown in the few studies that took this into consideration, this aspect has been largely ignored in most preclinical studies [9–11] as it is easier and cheaper to use only males [10, 11]. However, following the 2014 recommendations from NIH (National Institute of Health) to address this shortfall [12], more studies have investigated the role that sex may play.

A novel mechanism by which the dopamine system may modulate depression-like and anxiety-like behaviors in rats involves the dopamine D1-D2 receptor heteromer [13, 14]. This receptor complex has been identified in mouse, rat, monkey, and human striatum, using a variety of techniques such as co-immunoprecipitation [14–18], direct visualization using in situ FRET (Forster Resonance Energy Transfer) [17, 19–21] and in situ PLA (proximity ligation assay) [19, 21, 22]. Activation of D1-D2 heteromer led to anxiety-like [13] and depression-like [13, 14] phenotypes in male rats as detailed below. D1-D2 heteromer stimulation also blocked development of cocaine-induced conditioned place preference (CPP) and cocaine self-administration [19]. It also prevented development of locomotor sensitization to amphetamine [23] and cocaine [19], inhibited cocaine-induced ΔFosB accumulation, and phosphoERK activation [19]. Specific blockade of D1-D2 heteromer activity by a selective disrupting peptide TAT-D1 [14], reversed the abovementioned effects and revealed a tonic inhibitory role for the heteromer on the hedonic value of psychostimulant and natural rewards [13, 19, 21, 23].

Regarding the role of D1-D2 heteromer in depression and anxiety, we have previously shown that activation of the dopamine D1-D2 receptor heteromer by SKF 83959 induced depression-like and anxiety-like behaviors in male rats [13, 14]. Stimulation of D1-D2 heteromer by SKF 83959 significantly increased immobility and reduced the latency to immobility in the forced swim test (FST), which is commonly used as a measure of passive coping or behavioral despair [13, 14]. The elevated plus maze (EPM) test was also used to evaluate anxiety-like responses after D1-D2 heteromer stimulation [13]. In this test, SKF 83959-injected male rats spent less time in the open arms of the maze, denoting induction of an anxiogenic-like behavior. In addition, stimulation of D1-D2 heteromer by SKF 83959 abolished the willingness of trained animals to approach and consume sweetened milk in the novelty-induced hypophagia (NIH) test, which measures anxiety induced by the stress of a novel environment [13]. The involvement of the D1-D2 heteromer in all these SKF 83959-induced effects was established by the use of TAT-D1, a selective disrupting peptide for D1-D2 heteromer [14]. All the abovementioned behavioral effects were either attenuated, or reversed by pre-treatment with the TAT-D1 peptide prior to stimulation by SKF 83959. Thus, D1-D2 heteromer appears to contribute to depression-like and anxiety-related behavioral phenotypes in rodent models.

Since D1-D2 heteromer activation induced depression-like and anxiety-like behaviors, the present study was designed to investigate if any differences exist between D1-D2 heteromer density and functionality in male and female rats, focusing on differences in the signaling pathways postulated to be involved in mediating depression and anxiety. Higher levels of striatal D1-D2 heteromer in females versus males were confirmed in rats and a non-human primate model, suggesting that the sex difference in D1-D2 heteromer and its related functional effects observed in rat, likely exist in humans as similar differences in levels were reflected in monkey.

## Materials and methods

### Animals

Adult Sprague-Dawley rats (300–325 g; Charles River, Canada) were housed in pairs and maintained in a 12:12-h light:dark cycle with food and water available ad libitum. They were acclimatized for at least one week before inclusion in studies. Procedures were carried out in compliance with the guidelines in the Guide to the Care and Use of Experimental Animals (Canadian Council on Animal Care, 1993). The protocol was approved by the University of Toronto Animal Use Protocol Committee.

Adult African green monkey tissues (Chlorocebus sabeus, *n* = 3 males and 3 females) were acquired from Caribbean Primates, St. Kitts, as described [24]. All procedures were reviewed and approved by the Institutional Review Board of the Behavioural Sciences Foundation, St. Kitts, and the University of Toronto Animal Care Committee. All procedures were conducted according to the guidelines of the Canadian Council on Animal Care, the National Institutes of Health Guide for the Care and Use of Laboratory Animals, and the AVMA 2013 Guidelines on Euthanasia.

### Drugs

SKF 83959 hydrobromide (Tocris Bioscience) was dissolved in physiological saline containing 5% DMSO and administered subcutaneously. For non-drug injections, an equivalent volume of saline/vehicle was used. All drug injections were administered in a volume of 1.0 ml/kg. In rats that received the TAT-D1 peptide (Genscript; 300 pmoles/4 μl, i.c.v.), the drug or vehicle was administered 15 min prior to SKF 83959. The TAT-D1 peptide was dissolved in sterile water and diluted in physiological saline.

### Behavioral tests

#### Forced swim test

The forced swim test (FST) was conducted as described [14] in a non-colony room isolated from external noise. During the pre-test, animals were placed in a glass container with water at room temperature filled to a height of approximately 40 cm. Rats remained in the water for 15 min after which they were towel-dried and placed in a cage under a heat lamp until completely dry. Twenty-four hours following the pre-test, animals were administered vehicle, TAT-D1 peptide (300 pmol, i.c.v, administered 15 min pre-test) or SKF 83959 (0.1 mg/kg s.c., administered 5 min pre-test) and placed again in the water-filled container for 5 min. Immobility time and latency to immobility were measured.

#### Elevated plus maze

Testing was conducted as previously described [13] in an elevated plus maze (EPM) (Harvard Apparatus) situated in a non-colony room isolated from external noise. The EPM was constructed of black plexiglass and consisted of a central square with two sets of opposing open and closed arms each with dimensions 50 cm × 10 cm. Closed arms were enclosed by 40-cm high black plexiglass walls along the longitudinal edges, with the roof and ends open. The entire maze was suspended 50 cm off the ground. Following the assigned drug treatment of SKF 83959 (0, 0.1, 0.25 mg/kg, s.c.), rats were placed in the center of the maze and behavior was recorded for 10 min. Behavioral scoring of the videos occurred after the testing was complete and the following parameters were measured: time spent in the open arms, the number of open arm entries, and latency to first open arm entry. Entry to or exit from an arm was defined by both front paws crossing the arm boundaries. Behavioral testing took place 5 min following SKF 83959 injection.

### Surgeries

Rats were anesthetized with isoflurane (induction 5%, maintenance 2%), administered the analgesic Carprofen (5 mg/kg, s.c.) and secured in a stereotaxic frame. Body temperature was maintained at 37 °C by a warming pad. Custom electrode microarrays were built using pre-fabricated Delrin templates and polyimide-insulated stainless steel wires (A-M Systems: 791600, 0.008”) were implanted bilaterally into the NAc (AP + 1.9, ML ± 1.2, DV − 6.6 mm relative to bregma) and grounded by a reference wire attached to a screw fixed into the skull below lambda. Additional anchor screws were attached to the skull and electrodes secured with dental cement to the anchor screws. The animals received additional injections of Carprofen 24 and 48 h following surgery and were allowed to recover individually in their home cage for a minimum of 7 days before the experiments were performed. Electrode placement was validated post-mortem.

### Electrophysiology

All LFP oscillatory recordings were taken using a wireless system (W2100, Multichannel Systems) and were performed in awake, freely moving animals during EPM testing. Data were sampled at a rate of 1000 samples/second and the spectral power of LFP oscillations analyzed using routines from the Chronux software package for MATLAB (MathWorks). Recordings were downsampled, segmented, detrended, and low-pass filtered to remove frequencies greater than 100 Hz. Continuous multitaper spectral power (tapers = (5,9)) for each region was calculated for each segment in the following frequency bands: delta (1–4 Hz), theta (> 4–12 Hz), beta (> 12–32 Hz), slow gamma (> 32–60 Hz), and fast gamma (> 60–100 Hz).

### Co-immunoprecipitation of the D1-D2 heteromer

Co-immunoprecipitation was performed as previously described [14, 19]. Protein homogenates (250–300 μg) from rat NAc or CPu were incubated with an anti-D2R antibody (Alomone Laboratories) at 4 °C overnight under gentle rotation. After adding 40–50 μl of protein G/A, the mixture was further incubated for 1 h. After 3 washes with PBS-Tween, SDS buffer (70 μl) was added, and the immunoprecipitates were incubated for 5 min at 95 °C. Proteins were resolved by electrophoresis on 10% polyacrylamide gels under denaturing conditions (SDS-PAGE) and transferred onto nitrocellulose or PVDF membranes (Bio-Rad Laboratories, Hercules, CA, USA) using a semidry transfer system (Invitrogen, Carlsbad, CA, USA). Membranes were incubated in PBS-Tween (PBS-T)/10% nonfat milk for 1 h. After 3 washes, membranes were incubated with PBS-T/5% nonfat milk containing the anti-D1R antibody raised in rats (Sigma, St. Louis, MO, USA). Membranes were washed once in PBS-T and 2 times in PBS (10 min each) and incubated with the appropriate horseradish peroxidase (HRP)-conjugated polyclonal secondary antibody for 2 h. After 3 washes as indicated above, signal detection was performed using a chemiluminescence kit (Perkin-Elmer).

### Western blotting

Tissue corresponding to the NAc or CPu was collected from brains of each male or female rat (*N* = 6–9) rapidly after sacrifice. Following homogenization, 30–50 μg of protein from the indicated region were incubated in sample buffer for 3–5 min at 95 °C. Proteins were resolved by electrophoresis on 10% polyacrylamide gels under denaturing conditions (SDS–PAGE) and transferred onto nitrocellulose or PVDF membranes (Bio-Rad Laboratories, Hercules, CA) using a semidry transfer system (Invitrogen). Membranes were blocked in TBS-Tween (TBS-T)/5% nonfat milk for 1 h followed by incubation with PBS-T/5% nonfat milk containing the indicated first antibody overnight at 4 °C. Membranes were washed in TBS-T (3 × 10 min) and incubated with the appropriate horseradish peroxidase (HRP)-conjugated polyclonal secondary antibody (Bio-Rad) for 2 h at room temperature. After three washes as indicated above, signal detection was performed using a chemiluminescence kit (Perkin-Elmer). The primary antibodies used were anti-phosphoGSK-3 (1:1000, Cell Signaling), anti-BDNF (1:1000, Abcam), anti-phosphoTrkB (1:1000, Cell Signaling), and rabbit anti-GAPDH (1:10,000 or 1:20,000 Abcam).

### Proximity ligation assay (PLA)

In situ PLA was performed as described previously [19]. The PLA probes were created using a rat anti-D1R antibody (Sigma, D2944) conjugated with a PLUS oligonucleotide and a rabbit anti-D2R (Millipore, AB5084P) antibody with a MINUS oligonucleotide following manufacturer’s instructions (Duolink®, Sigma-Olink). The PLA protocol was performed as described by the manufacturer (Duolink®, Sigma-Olink). Briefly, coronal slices from rat brain (25 μm) or monkey (30 μm) were incubated for 1 h at 37 °C with the blocking solution in a pre-heated humidity chamber, followed by incubation with the generated PLA probes described above and washed with buffer A (DUO82047, Sigma-Olink). The PLA signal was detected using the Duolink II in situ PLA detection kit (DUO92008, Sigma-Olink) after the ligation-amplification steps. Nuclei were labeled by a DAPI solution included in the last washing step in buffer B × 0.01 (DUO82048, Sigma-Olink). Positive PLA signals were identified as red dots around the nuclei using Fluoview Olympus confocal microscope (FV 1000) with × 40/0.60 NA or × 60/1.2 NA objectives. Z-stacks were taken to confirm that PLA signals were localized on cell bodies. Three to four slices from each animal brain were used and at least four different images from each region were taken using × 60/1.2 NA. Each image dimension was 211.554 μm × 211.554 μm. Cell counting and analysis of the PLA signal were performed using Imagetool software (Duolink®). The reported percentages are calculated from images taken by the × 60/1.2 NA objective. Appropriate negative control assays were performed to ensure the specificity of the PLA labeling and amplification. Further controls using knock-out mice to validate the antibodies used were performed previously [14].

### Data analysis

For the FST time-course data, a repeated measures ANOVA with “time” as the within-subjects factors and “sex” as the between-subjects factor was used, followed by Bonferroni post-hoc tests. Analysis of the FST data with TAT-D1 was performed using a two-way ANOVA, followed by Bonferroni post-hoc tests as described in the text. The statistical significance of each dependent measure in the EPM was evaluated using a repeated measures ANOVA with a within-subjects factor of Dose and Sex as the between-subjects factor. For between subject comparisons at each dose, a Student’s *t* test was used. For the LFP data, the statistical significance of each dependent measure was evaluated using a repeated measures ANOVA with a within-subjects factor of Dose and Sex as the between-subjects factor. For planned between subject comparisons, a Student’s *t* test or paired Student’s *t* test was used as appropriate. The LFP spectral power from each group was normalized to the respective total spectral power taken during vehicle treatment. Quantification of the EPM measures, or LFP power data at each frequency, is reported as means ± sem. Planned comparisons were performed to evaluate within subject changes between SKF 83959 doses and vehicle (paired *t* tests), or to compare male and female rats at specific drug doses (*t* tests). Computations were performed using the SPSS/PC+ statistical package (IBM, Armonk, NY, USA).

For Western blots, to analyze the basal level difference between male and female rats, a two-way ANOVA was first applied using “sex” and “region” as between subjects, followed by a Bonferroni’s multiple comparisons test. A simple *t* test was performed using “sex” as between subjects for each region (NAc or CPu) when the ANOVA test was not appropriate. For the effect of treatment (control, SKF 83959, and SKF+TAT-D1), two approaches were used. The first one analyzed the effects on both sexes in the NAc, using a two-way ANOVA with “sex” and “treatment” as between subjects. The other approach analyzed the effect of treatment in each individual sex using a one-way ANOVA using “treatment” as within-subjects. The approach used for each analysis is indicated in the text describing the results. All data were expressed as means ± SEM.

## Results

### Dopamine D1-D2 receptor heteromer expression in male and female rats and monkeys

#### Co-immunoprecipitation and Western blot (WB)

**Fig. 1:**
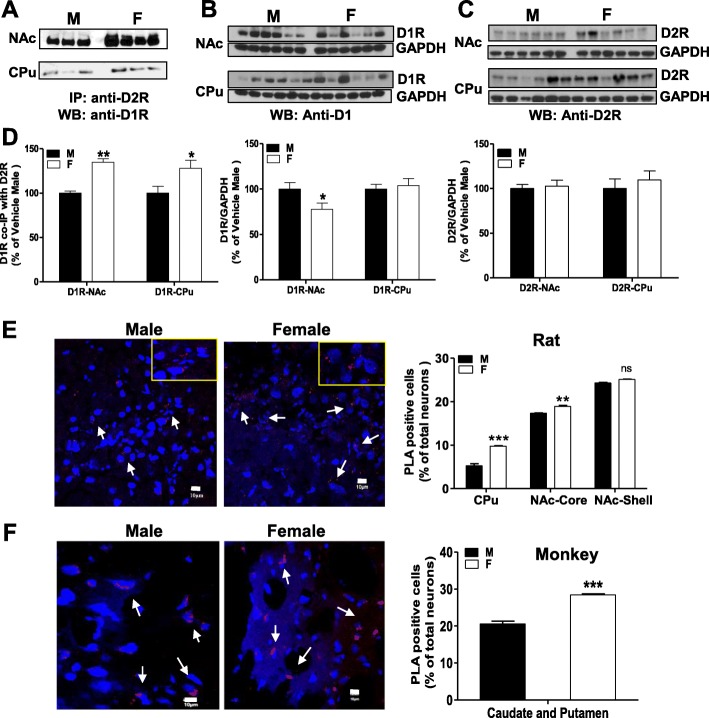
Comparison of the expression of dopamine D1-D2 receptor heteromer between male and female rats by co-immunoprecipitation (Co-IP) and proximity ligation assay (PLA). **a** Co-IP of D1R with anti-D2R antibody from NAc and CPu of male (M) and female (F) rats, followed by western blot to visualize D1R. **b** Western blot analysis of D1R in NAc and CPu in both sexes. **c** Western blot analysis of D2R in NAc and CPu of both sexes. **d** Quantification of Co-IP and WB results from **a**–**c**. *N* = 3–4 rats/group for Co-IP and *N* = 6 rats/group for WB. *p < 0.*05*, **p < 0.01 using *t* test. **e** Confocal images and insets (left) and quantification (right) of D1-D2 PLA signal in NAc and CPu of male and female rat. *N* = 6 rats/group, ANOVA, followed by Bonferroni’s post-test comparisons. **p < 0.*001*, ***p < 0.*0001*. Data are expressed as means ± SEM. **f** Confocal images (left) and quantification (right) of D1-D2 PLA signal in the caudate and putamen nuclei of male and female African Green monkey. *N* = 3 animals/group. ***p < 0.*0001* using *t* test. Data are expressed as means ± SEM

D1-D2 heteromer was immunoprecipitated by a specific D2 receptor (D2R) antibody followed by WB using a specific D1 receptor (D1R) antibody as described [14, 19]. Two-way ANOVA using “sex” and “region=NAc or CPu” as factors of variation showed a main effect of “sex” {*F*(1, 8) = 24.65, *p* = 0.001}, but not “region” {*F*(1, 8) = 0.29, *p* = 0.607}. Post-hoc tests showed that there was more D1R co-immunoprecipitated with D2R from both the nucleus accumbens (NAc, *p* < 0.01) and caudate-putamen (CPu, *p* < 0.05) of female rats than from male rats (Fig. 1a, d left panel). As in previous studies [14, 19], a mock control (IgG without tissue proteins) was used in parallel and no band was detected at the level of D1R, indicating the specificity of the co-immunoprecipitated D1R band (Supplementary Figure 1A).

Receptor expression estimated by simple WB revealed total D1R in NAc of male rats was significantly higher than in NAc of female rats (*t* test, *t* = 2.21 *p* = 0.042), with no apparent difference in CPu (*t* = 0.40, *p* = 0.694, Fig. 1b, d middle panel). D2R expression (Fig. 1c, d right panel) was not different between male and female rats in either region (*t* test, NAc: *t* = 0.35, *p* = 0.736; CPu: *t* = 0.67, *p* = 0.52). Taken together, this showed that female rats expressed a higher amount of D1-D2 heteromer in NAc and CPu and lower D1R in NAc than males, with no sex difference in D2R expression.

#### In situ proximity ligation assay (PLA)

In situ PLA showed that neurons positive for D1-D2 in rat striatum were highest in NAc shell (from *N* = 4452 and 3898 nuclei analyzed for male and female, respectively), followed by NAc core (from *N* = 5243 and 4188 nuclei analyzed for male and female, respectively) and lowest in CPu (from *N* = 2985 and 2294 nuclei analyzed for male and female, respectively), as detailed below (Fig. 1e). A two-way ANOVA revealed main effects of “sex” {*F*(1, 12) = 36.55, *p* < 0.0001} and “region” {*F*(1, 12) = 2929, *p* < 0.0001} and an interaction effect [sex × region] {*F*(2, 12) = 36.55, *p* < 0.0001}. Post-hoc analysis showed that female rats had a greater number of D1-D2 PLA-positive neurons than male rats both in NAc core (*p* < 0.001) and CPu (*p* < 0.0001) with comparable numbers in NAc shell (*p* > 0.05).

In monkeys (Fig. 1f), PLA analysis of D1-D2 expression in tissues from caudate and putamen nuclei (from *N* = 866 and *N* = 843 from females and males, respectively) showed females had a higher density of PLA-positive neurons (*t* test, *p* < 0.0001) expressing D1-D2 heteromer.

### Sex differences in behavioral tests of anxiety and depression in rats

#### Forced swim test

**Fig. 2:**
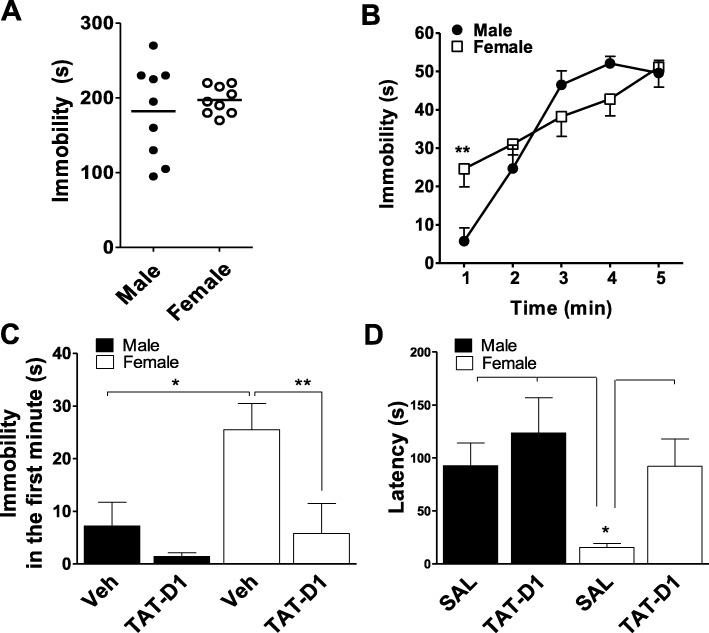
The dopamine D1-D2 heteromer mediates sex differences in pro-depressive responses in the forced swim test (FST). **a** In FST, no sex differences was observed for the total immobility time of the test. **b** Time-course of FST for male and female rats. Analysis showed a significant difference in immobility time only for the first minute of the test (**p<0.01). **c** Vehicle-treated female rats exhibited increased immobility in the first minute of testing compared to male rats (*p < 0.*05*). This difference was abolished by pre-treatment with TAT-D1 peptide (**p < 0.*01*). **d** The latency, in the first minute of FST, was highly reduced in vehicle-treated female rats compared to male rats, which was abolished by TAT-D1 (*p < 0.*05*). Error bars represent means ± S.E.M. *p < 0.*05*, **p < 0.*01*, ANOVA followed by Bonferroni’s post-hoc. *N* = 6 rats/group

Activation or disruption of the D1-D2 heteromer showed involvement in anxiety-like [13] and depression-like [13, 14] behaviors in adult male rats. To characterize whether the basal differential D1-D2 heteromer expression resulted in any innate sex differences in the susceptibility to depression-like behavior, responses in the forced swim test (FST) were evaluated (Fig. 2a–d). No sex difference was observed between vehicle-treated male and female rats for the total immobility time over the 5-minute test {*t* test, *t* = 0.69, *p* > 0.05} (Fig. 2a). However, analysis of the time-course (Fig. 2b) by a repeated measures ANOVA, showed that although no “sex” difference was observed {*F*(1, 50) = 0.46, *p* = 0.499}, there was a significant within-subjects “time” effect {*F*(4, 50) = 24.57, *p* < 0.0001}, as well as an interaction [sex × time] {*F*(4, 50) = 24.57, *p* = 0.009}. Post-hoc analysis revealed that the immobility was significantly different between male and female rats only during the first minute of the test (*t* = 3.16, *p* < 0.01; Fig. 2b). During this first minute of the FST (Fig. 2c), a Two-way ANOVA taking “sex” and “drug” as factors of difference showed that vehicle-treated female rats displayed increased immobility compared to males {main effect of sex: *F*(1, 20) = 6.6, *p* = 0.02}. Interestingly, this higher immobility in female rats was blocked by TAT-D1 peptide {main effect of drug: *F*(1, 20) = 8.3, *p* = 0.009, Fig. 2c}. Bonferroni’s post-tests showed that basal vehicle immobility values during the first minute were different between the sexes (*t* = 2.92, *p* < 0.05), and the values obtained with TAT-D1 pre-treatment were not different between sexes (*t* = 0.70, *p* > 0.05). Furthermore, these analyses also showed that the TAT-D1 effect was significant in female rats (*t* = 3.15, *p* < 0.05) but not in male rats (*t* = 0.93, *p* > 0.05), indicating D1-D2 heteromer involvement in the basal sex difference during the first minute of the FST test. These observations were then confirmed by investigating the latency to immobility during the first minute (Fig. 2d). A two-way ANOVA analysis using “sex” and “drug” as between-subjects indicated that vehicle-treated female rats showed a significantly lower latency than male rats {main effect of sex *F*(1, 20) = 5.3, *p* = 0.03}, which was reversed by pre-treatment with the TAT-D1 peptide {main effect of drug: *F*(1, 20) = 5.3, *p* = 0.03}. These data indicated that the basal difference in D1-D2 expression and activity mediated the sex difference in both the latency and immobility during the first minute of the FST. These FST results suggested that females were more susceptible than male to the pro-depressive effects of the basal activity of the D1-D2 heteromer, as assessed by TAT-D1 actions.

#### Elevated plus maze

**Fig. 3:**
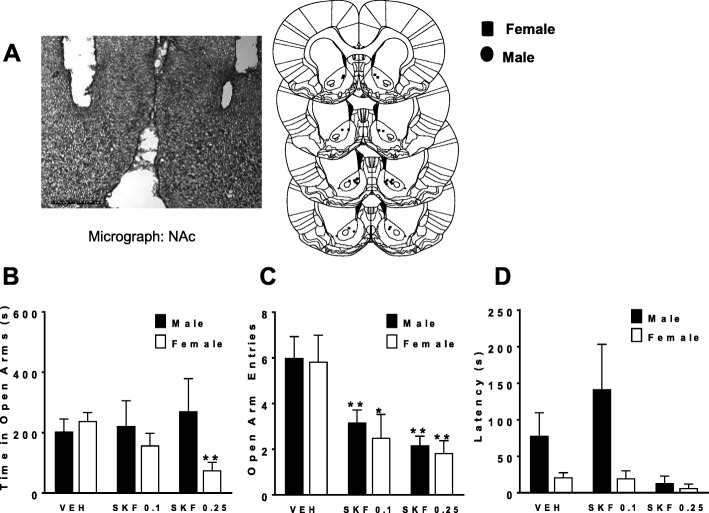
Increased female susceptibility to the anxiogenic effects of SKF 83959. Sex differences in susceptibility to low doses (0, 0.1, 0.25 mg/kg) of SKF 83959-induced anxiety responses were evaluated using the elevated place maze (EPM). **a** Representative micrograph, left, showing electrode placements, right, in NAc. **b** SKF 83959 (0, 0.1, 0.25 mg/kg, s.c.) dose-dependently reduced the total time spent in the EPM open arms in female, but not in male rats. **c** SKF 83959 decreased the number of open arm entries in both male and female rats. **d** SKF 83959 had no effect on latency to first entry. Error bars represent means ± SEM, *p < 0.*05*, **p < 0.*01*, compared to vehicle-treated rats of the same sex, paired Student’s *t* test. *N* = 5–6/group

**Fig. 4:**
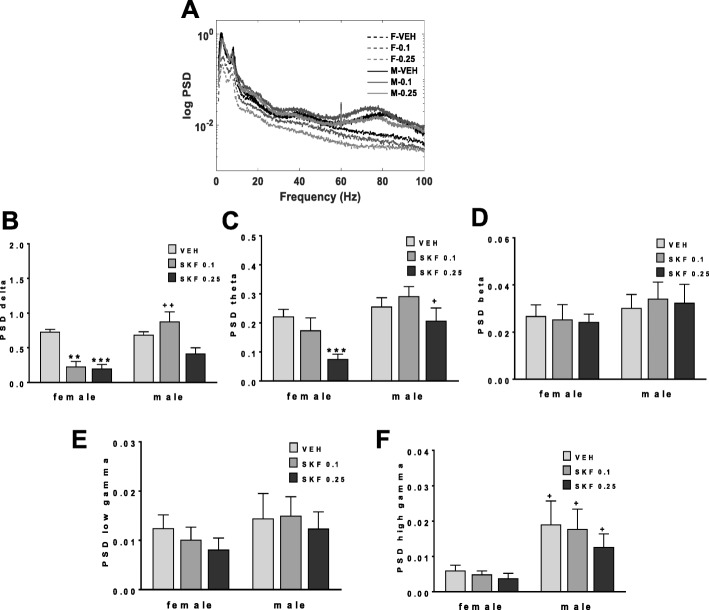
Sex difference in low-frequency oscillations recorded from NAc of animals exposed to SKF 83959. Local field potential (LFP) recordings from NAc were taken for the duration of each test for each animal subjected to the EPM after low doses (0, 0.1, 0.25 mg/kg) of SKF 83959 as indicated in Fig. 3. **a** Power spectra from male and female rats treated with vehicle or SKF 83959 (0.1 and 0.25 mg/kg). **b** Quantification of power spectra showing that female rats, but not male rats, exhibited a reduction in low-frequency delta in NAc in response to both doses of SKF 83959. **c** Quantification of power spectra showing a reduction in low-frequency theta oscillations in NAc of female rats only by 0.25 mg/kg SKF 83959. **d** Quantification of power spectra showing no sex difference in beta frequency oscillations in NAc. **e** Quantification of power spectra showing no sex difference in low-gamma frequency oscillations. **f** Quantification of power spectra showing innately lower NAc high gamma power in female rats compared to male rats. Error bars represent means ± SEM. ^+^*p < 0*.*05*, ^++^*p < 0*.*01* compared to female rats of the same dose, Student’s *t* test. **p < 0.*01*, ***p < 0.*001*, compared to vehicle-treated rats of the same sex, paired Student’s *t* test. *N* = 5–6/group with 2 electrodes/rat

**Fig. 5:**
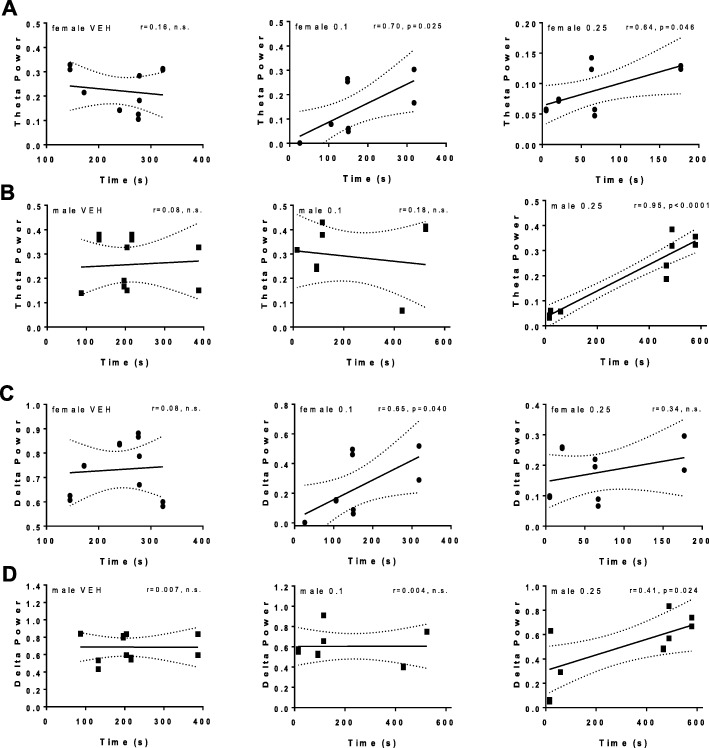
The anxiogenic effects of SKF 83959 are correlated with low-frequency oscillations in NAc. **a** Correlation curves showing a significant linear correlation between NAc theta oscillatory power with open arm time in female rats following 0.1 mg/kg and 0.25 mg/kg SKF 83959. **b** Correlation curves showing the relationship between NAc theta oscillatory power with open arm time in male rats following 0.1 and 0.25 mg/kg SKF 83959. A significant correlation was observed only at 0.25 mg/kg dose. **c** Correlation curves showing NAc delta oscillatory power with open arm time in female rats following 0.1 and 0.25 mg/kg SKF 83959. NAc delta oscillatory power correlated with open arm time in female rats selectively following 0.1 mg/kg SKF 83959. **d** Correlation curves showing the relationship between NAc delta oscillatory power with open arm time in male rats following 0.1 and 0.25 mg/kg SKF 83959. NAc delta oscillatory power correlated with open arm time in male rats selectively following 0.25 mg/kg SKF 83959. Points represent values taken from individual electrodes with line of best fit shown. *N* = 5–6/group with two electrodes/rat

To evaluate sex differences in susceptibility to D1-D2-induced anxiety responses, different SKF 83959 doses (0, 0.1, 0.25 mg/kg) were administered to male and female rats every 48 hours in a repeated measures design followed by testing in the elevated place maze (EPM; Fig. 3) as described [13]. As neural oscillations have physiological patterns that are highly conserved across species and, importantly, are coupled to specific behavioral states, we wanted to evaluate whether there was a link between oscillations recorded from NAc and SKF 83959-induced effects on anxiety (Figs. 4 and 5). Local field potential (LFP) recordings from NAc were taken for the duration of each EPM test, with placements shown (Fig. 3a). In a previous study, SKF 83959 showed a lack of anxiogenic effect at doses lower or equal to 0.5 mg/kg in male rats subjected to EPM [13]. In the present EPM data, repeated measures ANOVA revealed a significant interaction [sex × SKF Dose] for total open arm time {*F*(2, 20) = 3.9, *p* = 0.038; Fig. 3b} and a main effect of SKF Dose for open arm entries {*F*(2, 20) = 31.2, *p* < 0.0001; Fig. 3c}. Female rats exhibited dose-dependent reduction in time spent in the open arms with no effect of these SKF 83959 doses in the male rats (Fig. 3b). Both male and female rats showed reduced open arm entries with either dose of SKF 83959 (Fig. 3c) with no effects on latency to first entry (Fig. 3d).

LFP recordings from NAc (Fig. 4) showed a significant main effect of “sex” on the low-frequency oscillations, delta {*F*(1, 19) = 15.7, *p* < 0.0001; Fig. 4b}, theta {*F*(1, 19) = 5.5, *p* = 0.03; Fig. 4c}, and high gamma {*F*(1, 19 ) = 7.8, *p* = 0.012; Fig. 4f}, but no sex difference in beta (Fig. 4d) and low gamma (Fig. 4e) frequencies. It also showed a within-subjects effect of SKF Dose for delta {*F*(2, 38) = 8.5, *p* = 0.001} and theta {*F*(2, 38) = 13.1, *p* < 0.0001}, and a [sex × SKF Dose] interaction for delta {*F*(2, 38) = 5.2, *p* = 0.01}. Female rats innately exhibited reduced high gamma power in NAc compared to males (*p* = 0.050, Student’s *t* test; Fig. 4f) but were not otherwise different from male rats. However, low-frequency responses to SKF 83959 were different, with females exhibiting significantly reduced delta (*p* < 0.0001 vs vehicle, paired *t* test) and theta power (*p* < 0.0001 vs vehicle, paired *t* test) following 0.25 mg/kg of SKF 83959; effects that were not evident in male rats (Fig. 4b, c). To further understand the relationship between NAc low-frequency oscillations and anxiety in the EPM, regression analyses were performed between theta (Fig. 5a, b) or delta (Fig. 5c, d) power with EPM open arm time for each sex. No linear correlation for either sex was evident following vehicle treatment. With administration of SKF 83959 0.1 mg/kg, both theta (*r* = 0.70, *p* = 0.025, Fig. 5a) and delta (*r* = 0.65, *p* = 0.040, Fig. 5c) power were significantly correlated with Open Arm Time in female, but not male rats (Fig. 5b, d). Following SKF 83959 2.5 mg/kg, theta power was correlated to Open Arm time in females (*r* = 0.64, *p* = 0.046, Fig. 5a), whereas correlations with both theta (*r* = 0.95, *p* < 0.0001, Fig. 5b) and delta (*r* = 0.64, *p* = 0.024, Fig. 5d) power were evident in male rats. Together these findings indicate that female rats were more susceptible to the anxiogenic effects of SKF 83959, further highlighting a potential relationship between D1-D2 activation, anxiety levels, and low-frequency oscillations in NAc.

#### Signaling pathway differences between male and female rats

Several proteins implicated in anxiety and/or depression are differentially regulated between female and male rodents in the hippocampus, amygdala and/or medial prefrontal cortex (mPFC) [25]. Some of these proteins are also modulated by the D1-D2 heteromer signaling pathway, such as BDNF, its receptor TrkB [17, 26], ERK [19] and GSK3 [26]. Alterations of these proteins were compared in male and female rats at the basal level and after heteromer activation with SKF 83959 (0.4 mg/kg s.c. for 5 days). Involvement of the D1-D2 heteromer in the SKF 83959 effects was confirmed by pre-treatment with heteromer disrupting TAT-D1 (300 pmol i.c.v., 5 days). The following experiments show results from adult female rats compared to adult male rats (*n* = 6–9 per group). We analyzed the basal level of each protein involved in both the NAc and CPu of both male and female animals. However, after treatment, only the NAc was analyzed as the CPu expressed much lower D1-D2 heteromer levels than the NAc.

### BDNF/pTrkB

#### BDNF

**Fig. 6:**
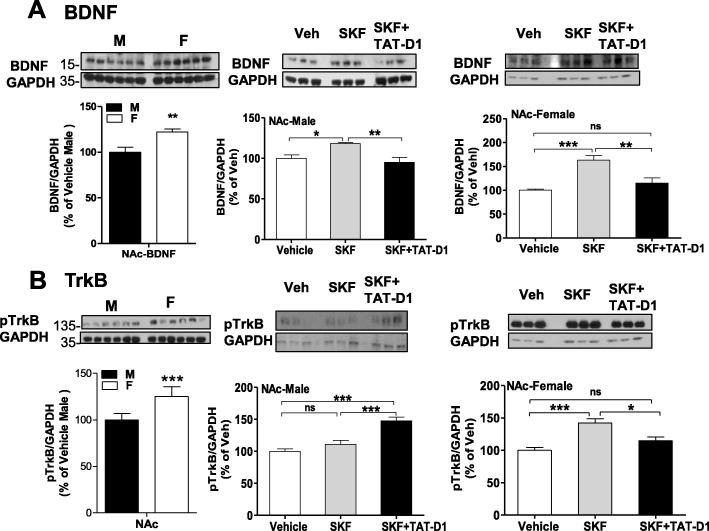
Differences between male and female rats in the BDNF/TrkB signaling pathway. Western blot analysis of BDNF (**a**) and phospho-TrkB (**b**) in male and female rats. **a** In control rats treated with vehicle (left panel), the basal level of BDNF expression was significantly higher in the NAc of female compared to male rats (*p < 0*.*05*). Repeated treatment with SKF 83959 (0.4 mg/kg s.c. × 5 days) significantly increased BDNF expression in the NAc of male rats (middle panel; *p = 0*.*011*), which was blocked by pre-treatment with TAT-D1 peptide (300 pmol i.c.v.; *p < 0*.*05*). Similar effects were observed in female rats (right panel; *p = 0*.*0003*). *N* = 6 rats/group. Data are means ± SEM. **b** A trend to higher phosphorylation level of TrkB (pTrkB) in the NAc of female rats compared to male rats was noted (left panel), but no statistical difference was observed (unpaired *t* test, *p = 0*.*54*). Treatments had a significant effect on pTrkB in the NAc (middle panel) of male rats (ANOVA; *p* = <0.0001), SKF 83959 treatment (0.4 mg/kg s.c. × 5 days) had no significant effect (*t = 1*.*43*), whereas TAT-D1 peptide pre-treatment (SKF59+TAT-D1) was significantly higher than both vehicle and SKF 83959 treatment. In female NAc (right panel), a clear effect of treatment was observed (ANOVA; *p = 0*.*0003*), with SKF 83959 increasing pTrkB in comparison to vehicle (*p < 0*.*05*), whereas pre-treatment with TAT-D1 blocked the effect of SKF 83959 (*p < 0*.*05*). *N* = 6 rats/group. Data are means ± SEM

In control vehicle-treated rats (Fig. 6a, left panel), our preliminary analysis taking solely “sex” as a factor of variation showed that basal BDNF expression was significantly higher in NAc of female compared to male rats {one-way ANOVA, *F*(1, 13) = 7.714, *p* < 0.05}. We next analyzed the effects of treatments with SKF 83959, in the presence or the absence of TAT-D1 peptide, within each sex. Repeated treatment with SKF 83959 significantly increased BDNF in the NAc of male rats (Fig. 6a middle panel; {one-way ANOVA, *F*(2, 17) = 6.137, *p* = 0.011}), which was blocked by pre-treatment with TAT-D1 (post-hoc, *t* = 3.35, *p* < 0.05, SKF vs SKF+TAT-D1). Similar effects were observed in female rats {one-way ANOVA, *F*(2, 15) = 14.93, *p* = 0.0003}, with SKF 83959 increasing BDNF expression (post-hoc: *t* = 5.23, *p* < 0.05, vehicle versus SKF), which was blocked by TAT-D1 (post-hoc: *t* = 3.98, *p* < 0.05, SKF vs SKF+TAT-D1) (Fig. 6a, right panel). To confirm these results, we also used another approach of analysis, which included both “sex” and “treatment” as factors of variation. This two-way ANOVA analysis showed that there was a “sex” effect {*F*(1, 30) = 15.27, *p* = 0.0005}, a “treatment” effect {*F*(2, 30) = 21.16, *p* < 0.0001} and an interaction [sex × treatment] {*F*(2, 30) = 5.495, *p* = 0.0093}. It was noted from Bonferroni’s post-tests analysis that the SKF 83959-induced BDNF increase was greater (*t* = 6.56, *p* < 0.001) in NAc of female than in male rats. In contrast, no significant effect was observed in the CPu, with no “sex” effect {*F*(1, 30) = 3.92, *p* = 0.057}, no “treatment” effect {*F*(2, 30) = 3.23, *p* = 0.054} and no interaction [sex × treatment] observed {*F*(2, 30) = 1.543, *p* = 0.230}.

#### phosphoTrkB

We then analyzed the phosphorylation of the BDNF receptor, TrkB (pTrkB) at basal levels taking account of “sex” and “region = NAc and CPu” as factors for variations (Fig. 6b, left panel). A two-way ANOVA analysis showed that there was a “sex” effect {*F*(1, 23) = 20.15, *p* = 0.0002}, a “region” effect {*F*(1, 23) = 16.44, *p* = 0.0005}, and an interaction [sex × region] {*F*(1, 23) = 16.44, *p* = 0.0005}. Bonferroni’s post-tests showed that there was a significant sex difference in basal pTrkB in the NAc (*t* = 6.33, *p* < 0.001; Fig. 6b, left panel), but not in the CPu (*t* = 0.29, *p* > 0.05).

The effects of treatment with SKF 93959, in the presence or the absence of TAT-D1 peptide were investigated in the NAc of male and female rats (Fig. 6b, middle and right panels, respectively). A first analysis using two-way ANOVA, with “sex” and “treatment” as factors of variation showed that “sex” did not affect the results {*F*(1, 30) = 0.01, *p* = 0.914}, whereas “treatment” had a significant effect on the results {*F*(2, 30) = 18.48, *p* < 0.0001}, with an interaction [sex × treatment] {*F*(2, 30) = 16.60, *p* < 0.0001}. Bonferroni’s post-tests analysis showed that SKF 83959 had an effect in female (*t* = 5.38, *p* < 0.001) but not in male NAc (*t* = 1.40, *p* > 0.05), in contrast to TAT-D1 + SKF 83959 treatment which had a significant effect in male NAc (*t* = 6.07, *p* < 0.001) but had no effect on pTrkB in female NAc (*t* = 1.90, *p* > 0.05).

To confirm these data, we also analyzed the effect of “treatment” alone on each individual sex (Fig. 6b, middle and right panels, respectively). Treatments had a significant effect in male rat NAc pTrkB (one-way ANOVA {*F*(2, 15) = 21.14, *p* < 0.0001}, with Bonferroni’s post-tests showing SKF 83959 had no effect compared to vehicle (*t* = 1.43, *p* > 0.05) whereas pre-treatment with TAT-D1 increased pTrkB compared to vehicle (*t* = 6.21, *p* < 0.001) and compared to SKF 83959 alone (*t* = 4.78, *p* < 0.001). In female NAc (Fig. 6b, right panel), a clear effect of treatment was observed {*F*(2, 15) = 14.24, *p* = 0.0003}, with SKF 83959 increasing pTrkB compared to vehicle (*t* = 5.23, *p* < 0.05), which was blocked by TAT-D1 (*t* = 3.99, *p* < 0.05).

### Akt/GSK3/beta-catenin signaling

#### GSK3

**Fig. 7:**
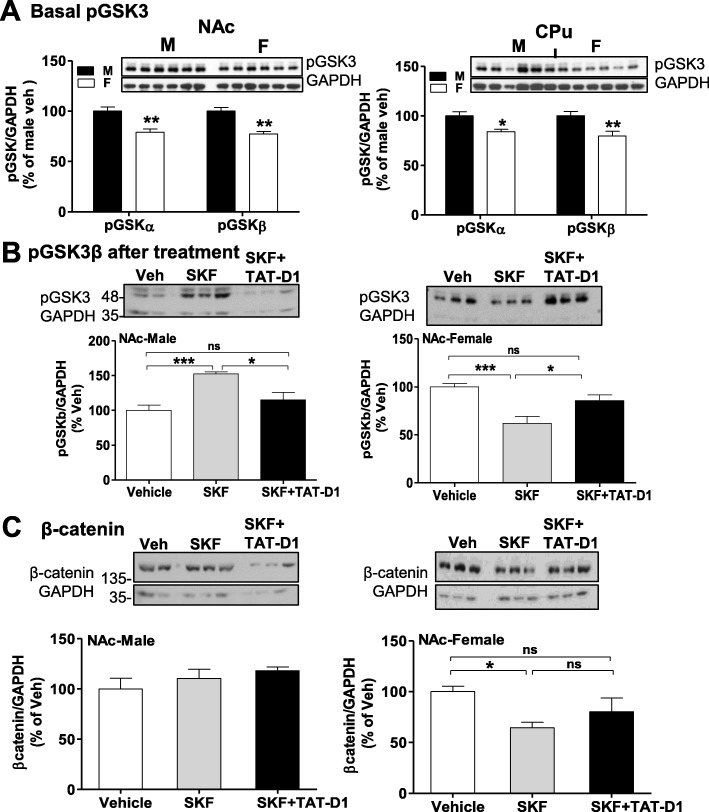
Differences between male and female rats in the Akt/GSK3/β-catenin signaling pathway. Modulation of GSK3/β-catenin. Western blot analysis of phospho-GSK3 (pGSK, **a**, **b**), β-catenin (**c**) and phospho-Akt (pAkt) at two sites, threonine 743 (pThr743-Akt, D, left panel) and serine 308 (pSer308-Akt, E, right panel) in male and female rats. These animals were treated for five days with vehicle, SKF 83959 (SKF59) or pre-treated with TAT-D1 followed with SKF 83959 (SKF59 + TAT-D1). *N* = 6/group. **a** In the NAc, the basal phosphorylation of both isoforms of GSK3, pGSKα (*p < 0*.*01*), and pGSKβ (*p < 0*.*01*) were lower in female compared to male rats. Similar results were observed in the CPu (*p < 0*.*01*). **b** In the NAc of male rats (7b, left panels) repeated treatment with SKF 83959 (0.4 mg/kg × 5 days) increased pGSKβ in comparison to vehicle-treated rats (*p < 0*.*005*), which was inhibited by pre-treatment with TAT-D1 peptide (*p < 0*.*05*). In the NAc of female rats (5b, right panel), SKF 83959 induced a significant decrease in pGSKβ (*p < 0*.*05*), which was blocked by pre-treatment with TAT-D1 (*p < 0*.*05*). **c** In male rats (7c, left panel), treatment with SKF 83959 alone or after pre-treatment with TAT-D1 had no significant effect on β-catenin expression in the NAc (*p < 0*.*05*). In the NAc of female rats (7c, right panel), SKF 83959 decreased β-catenin expression (*p > 0*.*05*)

The basal phosphorylation of both isoforms of glycogen synthase kinase-3 (GSK3), pGSK3α, and pGSK3β, were analyzed by WB in NAc and CPu of male and female rats (Fig. 7a). A Two-way ANOVA analysis taking account the “isoforms” and the “sex” as factors of variation showed a main effect of sex {*F*(1, 20) = 41.52; *p* < 0.0001}in the basal phosphorylation of both isoforms, with both pGSKα (post-hoc: *t* = 4.38, *p* < 0.01) and pGSKβ (*t* = 4.73, *p* < 0.01) showing decreased phosphorylation in female NAc compared to male rats (Fig. 7a). Similar results were observed by analyzing the basal phosphorylation of the two isoforms in the CPu {two-way ANOVA; *F*(1, 20) = 19.27, *p* < 0.0001}. Both orthologues of GSK3 were modulated identically by the different treatments as shown below, for this reason, only variations in pGSKβ will be described in the following section (Fig. 7b). Also, and since the basal level of phosphorylation was different between male and female rats, the effect of treatments was analyzed within-subjects for each individual sex separately.

In NAc of male rats, One-way ANOVA analysis showed an effect of “treatment” on pGSKβ {*F*(2, 18) = 7.40, *p* = 0.0045} (Fig. 7b, left panel). Post-hoc tests showed that treatment with SKF 83959 increased pGSKβ in comparison to vehicle treatment (*t* = 3.71, *p* < 0.01), and this SKF 83959 effect was inhibited by pre-treatment with TAT-D1 peptide (*t* = 2.89, *p* < 0.05). In contrast, in NAc of female rats (Fig. 7b, right panel) analysis {one-way ANOVA, *F*(2, 15) = 11.29, *p* = 0.001} showed that SKF 83959 induced a significant decrease in pGSKβ (post-hoc: *t* = 4.70, *p* < 0.05, Veh vs SKF), which was blocked by TAT-D1 (post-hoc: *t* = 2.94, *p* < 0.05, SKF vs SKF+TATD1). Thus, pGSKβ was more active (i.e. less phosphorylated) under basal conditions in female NAc than in male rats, and furthermore, it was differentially modulated by the heteromer in a sex-dependent manner.

#### β-catenin

To evaluate downstream effects, a target of GSK3 action, β-catenin [27] was investigated (Fig. 7c). A two-way ANOVA using “sex” and “region” as factors of variations of basal β-catenin expression showed that there was no significant effect of sex {*F*(1, 20) = 0.322, *p* = 0.5768}, or region {*F*(1, 20) = 3.28, *p* = 0.0852}, suggesting there was no significant difference in the basal expression of β-catenin between male and female rats in either region analyzed, NAc or CPu.

We analyzed the effect of drug treatment on the β-catenin levels in the NAc of each sex individually. In male rats (Fig. 7c, left panel), treatment with SKF 83959 alone or after pre-treatment with TAT-D1 had no significant effect on β-catenin expression in NAc (One-way ANOVA, {*F*(2, 17) = 1.151, *p* = 0.343}). In contrast, there was an effect of treatment in female NAc (One-way ANOVA, {*F*(2, 22) = 4.210, *p* = 0.028}, Fig. 7c, right panel). SKF 83959 decreased β-catenin expression in the NAc of female rats (Veh versus SKF, *t* = 2.89, *p* = 0.017), which was partially blocked by TAT-D1 (*t* = 1.25, *p* > 0.05, Veh versus SKF + TAT-D1), indicating an effect mediated in part by the D1-D2 heteromer.

#### Akt

**Fig. 8:**
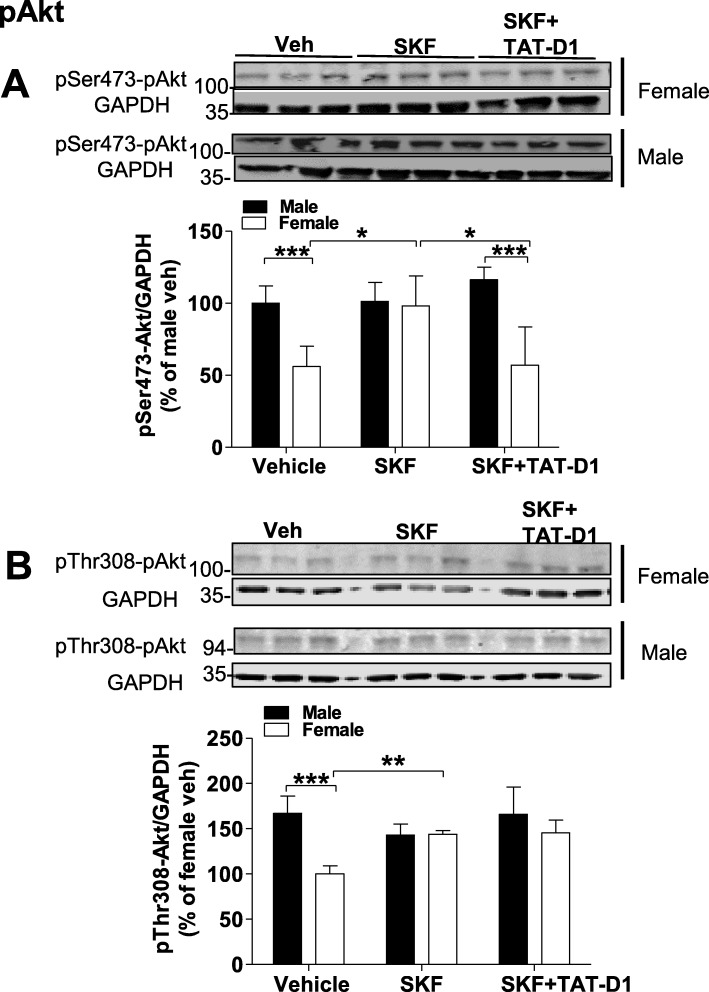
Differences between male and female rats in the Akt/GSK3/β-catenin signaling pathway. Modulation of Akt. Phosphorylation of Akt at Ser473 (**a**) and Thr308 (**b**). At basal level, Akt was more active (more phosphorylated) in male than in female (*p < 0*.*0001*) for both sites. SKF 83959 increased the phosphorylation of Akt on both sites, pThr308-Akt (**a**) and pSer473-Akt (**b**), in female rats. Pre-treatment with TAT-D1 peptide blocked the SKF 83959 effects on pSer473 but had no notable effect on pThr308 site in female rats. No effect of treatment was observed in the NAc of male rats. *N* = 6 rats/group. Data are means ± SEM

Of the kinases upstream of GSK3, Akt modulates GSK3 activity with powerful effects within the dopamine system (reviewed, [27, 28]). Multiple sites of phosphorylation of Akt exist but the two major sites important for its activity are Ser473 and Thr308 (reviewed, [27]). Phosphorylation at these sites was evaluated in the basal state and after the treatments. At the basal level, Akt had higher activity (more phosphorylated) in male NAc than in female {*F*(1, 20) = 65.07, *p* < 0.0001} at both sites (Fig. 8). Statistical analysis using two-way ANOVA and “sex” and “treatment” as factors of variation showed that for pSer473-Akt (Fig. 8a), there was an effect of sex {*F*(1, 30) = 39.54, *p* < 0.0001}, of treatment {*F*(1, 30) = 5, *p* = 0.0134}, and an interaction [treatment × sex] {*F*(1, 30) = 8.81, *p* = 0.001}. Similarly, for pThr308-Akt (Fig. 8b) there was an effect of “sex” {*F*(1, 30) = 27.75, *p* < 0.0001}, of “treatment” {*F*(1, 30) = 5.45, *p* = 0.0096} and an interaction [treatment × sex] {*F*(1, 30) = 13.29, *p* < 0.0001}. Bonferroni’s post-test analyses showed that SKF 83959 had no effect on male pSer473 (*t* = 0.14, *p* > 0.05) nor pThr308 (*t* = 0.09, *p* > 0.05). In contrast, SKF 83959 increased the phosphorylation of Akt at both sites (pSer473: *t* = 4.36, *p* < 0.001; pThr308: *t* = 4.61, *p* < 0.001) in female rats to levels equivalent to those in male rats (SKF-male versus SKF-female, pSer473: *t* = 3.26, *p* > 0.05; pThr308: *t* = 0.09, *p* > 0.05). Pre-treatment with TAT-D1 in female rats blocked SKF 83959 effect on pSer473 (SKF versus SKF+TAT-D1, *t* = 4.20, *p* < 0.001) but had no significant effect on SKF 83959-induced increase in the phosphorylation of pThr308 (SKF versus SKF + TAT-D1, *t* = 0.15, *p* > 0.05). Thus, Akt was more active in the basal state in male rat NAc than in female, which would explain the sex difference observed in GSK3 phosphorylation at the basal level. These results also indicated that D1-D2 heteromer was differentially involved in the modulation of the Akt-Ser473 site versus the Thr308 site, at least in female rat NAc.

## Discussion

The present study showed a significant sex difference in the expression of the dopamine D1-D2 receptor heteromer in rat NAc and CPu and in non-human primate caudate nucleus, with females found to express a higher density of D1-D2 complexes with a greater number of heteromer-expressing neurons compared to males. The higher heteromer density and number of neurons expressing the D1-D2 heteromer in female rats occurred despite the fact that D1 receptor expression was lower in female than in male rats, with no difference in D2 receptor expression between the two sexes. The results also indicated that female rats showed greater susceptibility to the pro-depressive and anxiogenic-like effects related to the basal activity of D1-D2 heteromer as assessed in the forced swim test, as well as after its activation by low doses of SKF 83959 as assessed in the forced swim and EPM tests, all of which were ameliorated by disruption of the heteromer by the selective peptide TAT-D1. Sex differences in the EPM correlated with differences in LFP recordings from NAc, notably at the low-frequency delta and theta oscillations. These results suggest that higher D1-D2 heteromer expressed in female rat and female non-human primate may have greater potential for inducing aversion, shown to be associated with heteromer activation [19]. Furthermore, lower D1 receptor expression may suggest overall lower reward reinforcement in females, suggesting this combination of reduced reward and increased aversive function could significantly increase predisposition to depressive-like behavior in females.

Indeed, the behavioral studies showed that the difference in D1-D2 heteromer expression was involved in the sex-based difference in greater susceptibility to anxiogenic and pro-depressive-like behavior by basal activity of the heteromer as well as heteromer activation using low doses of SKF 83959. Higher SKF 83959 doses were necessary to induce similar effects through D1-D2 heteromer activation in male rats in the FST and EPM [13, 14, 23]. Although no sex difference in total immobility time in FST was observed, time-course analysis revealed that vehicle-treated female rats exhibited increased immobility early in the first minute, which was abolished by TAT-D1 peptide. Further, the latency to immobility was dramatically lower in female rats compared to male rats, a difference also abolished by TAT-D1 peptide. These results indicated that female rats were more susceptible to the pro-depressive basal activity of D1-D2 heteromer and this translated by earlier and faster immobility than male rats. Moreover, in the EPM paradigm, in which male rats were found susceptible to the anxiogenic effects of higher doses of SKF 83959 (more than 0.5 mg/kg) through D1-D2 activation as evidenced by its reversal by TAT-D1 [13], female rats showed greater sensitivity and dose-dependent susceptibility than male rats to the anxiogenic-like effects of low doses of SKF 83959. Taken together, these behavioral data indicated that the higher D1-D2 heteromer expression in female than in male rats may be a reason for the susceptibility of female rats to the pro-depressive and pro-anxiogenic effects of the basal as well as activated state of the heteromer.

LFP recordings from the NAc of rats subjected to EPM showed a significant sex difference at the low-frequency delta and theta oscillations, as well as in high-frequency gamma oscillations at the basal level. Responses to low doses of SKF 83959 also showed a sex difference with females demonstrating reduced delta and theta oscillations in contrast to a lack of effect in male rats. Further, the spectral power of these oscillations was correlated to time spent in the open arms following SKF 83959 treatment. The behavioral tests, taken together, confirm that the D1-D2 heteromer is highly involved in depression-like and anxiogenic-like behaviors and highlight a potential relationship between anxiety level and low-frequency oscillations in NAc.

Signaling pathways were investigated due to their postulated contribution to anxiety and/or depression [25, 29]. Although the focus has been on alterations in PFC, hippocampus, and amygdala, there is strong evidence for a key role of the mesolimbic system in their modulation [25, 29]. For example, BDNF/TrkB signaling has opposite effects on depression and anxiety depending on the brain region investigated. In mesolimbic regions, an increase in BDNF was pro-depressive, in contrast to the anti-depressant effects of BDNF in PFC and hippocampus [25, 29]. Activation of the D1-D2 heteromer increased BDNF production in cultured striatal neurons and in NAc of male rats [17, 26], with pro-depressive [13, 14] and anxiogenic behavioral effects [13]. In contrast, central administration of the D1-D2 disrupting peptide, TAT-D1 resulted in anti-depressant-like effects in male rats exposed to chronic unpredictable stress [13], a model believed to better predict the therapeutic power of antidepressants in chronic depressive-like conditions [30]. In the present study, female rats expressed higher basal level of BDNF and activated phosphorylated TrkB in NAc, which could be related to higher D1-D2 heteromer expression and activity in females. Repeated injections of SKF 83959 further increased BDNF expression and TrkB activation in female rats and to a lesser extent in male rats, effects that were inhibited by TAT-D1. These observations indicate that basal BDNF/TrkB signaling activity is more pronounced in NAc of female rat compared to male, with D1-D2 heteromer activation leading to increased BDNF/TrkB signaling in both sexes, but more prominently in female rats. This difference may account for higher susceptibility in female rats to pro-depressive- and anxiogenic-like behavior in the basal state, further stimulated by D1-D2 heteromer activation compared to male rats.

An important downstream target of BDNF/TrkB signaling is the Akt/GSK3 pathway. Optimal activation of Akt is critically dependent on phosphorylation at Ser473 and Thr308. Akt phosphorylation at Ser473 and Thr308 were lower in female rat NAc than in male, demonstrating a significant sex difference in the basal activation state of Akt. Interestingly, lower phosphorylation of GSK3 isoforms was observed in female rats, suggesting a causal relationship to the lower Akt phosphorylation, with resultant increased GSK3 activity in female rats comparatively to male rats. Whether this sex difference in basal Akt activity is linked to D1-D2 heteromer activity was not directly explored, but the blockade by TAT-D1 of SKF 83959-induced increase of pAkt in female rats may suggest that this signaling pathway may be, at least in part, under the influence of the heteromer. In fact, SKF 83959 increased the activity of Akt in female rats by phosphorylation at Thr308 and Ser473 to an extent exhibited by male rats at the basal level. However, this increase in pAkt in female rats did not translate to an increase in pGSK3, since SKF 83959 further decreased GSK3 phosphorylation, suggesting the effect of SKF 83959 on GSK3 in female rat NAc may be not mediated through the Akt pathway. Activation of the dopamine D2 receptor (D2R) leads to dephosphorylation and inactivation of Akt [31, 32], through a complex consisting of βarrestin2-Akt-protein phosphatase 2A [βarr2-Akt-PP2A] [33], which results in activation and reduced phosphorylation of GSK3 [31–33]. Further, GSK3 is constitutively active, and its inhibition by phosphorylation can also be mediated by other kinases, such as PKA, PKC, CaMKII, CDK5 (reviewed, [27, 34]). Modulation of GSK3 activity, notably its β subunit, through the βarr2-Akt-PP2A complex is mediated essentially through D2R, to a lesser extent through D3R and did not involve D1R directly [31, 33, 35, 36]. However, activation of D1R and D2R in primary neuronal cultures [37] or D2R in transfected cells [38] can activate Akt leading to GSK3β inhibition. Interestingly, SKF 83959 treatment led to increased Akt phosphorylation and inhibition of GSK3 activity through activation of the D1-D2 heteromer or D5R in the PFC of male rats [26]. The mechanisms involved in PFC were different, with the D1-D2 heteromer effect independent of BDNF/TrkB unlike the D5R effect. In rat NAc, activation of BDNF/TrkB occurred through repeated stimulation of D1-D2 heteromer in both sexes, but the consequences on GSK3 activation showed sexual dichotomy, mirrored by the differential modulation of its phosphorylation and the accumulation of one of its substrates, β-catenin.

Regarding the sex difference observed here, our hypothesis is that in the basal state, due to higher D1-D2 heteromer and lower D1R expression in NAc of female rat, the predominant effect on Akt/GSK3 may be through the D2R medium spiny neurons (D2-MSN) via βarr2-Akt-PP2A [31, 32], whereas in male rat the basal activity of Akt/GSK3 would also be governed by PI3K signaling in D1-MSNs. In female rat NAc, where D1 receptors are lower, BDNF/TrkB action in D1-MSNs would be counterbalanced by the effect exerted by the D2-MSNs through βarr2-Akt-PP2A. This could explain the sex difference in basal phosphorylation of both Akt and GSK3, i.e., lower phosphorylation of Akt resulting in lower phosphorylation of GSK3 in female NAc compared to male. Thus, activation of D1-D2 heteromer would increase BDNF release, activating TrkB receptors localized on both D1- and D2-MSNs [39] with different outcomes in male and female rats.

This hypothesis could represent a first indication that D1-D2 heteromer activation through BDNF and TrkB signaling could be one key player in the modulation of the two well-characterized D1-MSN and D2-MSN pathways. Further experiments are needed to shed more light on the importance of the D1-D2 heteromer in modulating D1-MSNs and D2-MSNs.

The sex difference in the modulation of phosphorylation and activity of GSK3 was accompanied by a sex difference in the accumulation of β-catenin. D1-D2 heteromer activation in female rats decreased GSK3 phosphorylation resulting in decreased β-catenin. Activation of GSK3 leads to β-catenin degradation, whereas GSK3 inhibition leads to accumulation of β-catenin and translocation to the nucleus, where it affects the expression of multiple genes [39]. In a model of chronic social defeat stress, β-catenin activity was reduced in the NAc of susceptible mice, whereas resilient mice showed enhanced β-catenin activity [39, 40]. Reduced β-catenin activity was also documented in NAc of depressed humans [39]. Furthermore, the effects of β-catenin were observed selectively through overexpression in D2-MSNs [39] and not in D1-MSNs, which led to a pro-resilient effect whereas local knockdown or expression of a β-catenin dominant negative construct led to enhanced susceptibility to stress [39]. The sex difference in our model would fit with heightened activation of GSK3 through lower phosphorylation due to activation of the complex βarr2-Akt-PP2A in D2-MSNs, leading to decreased β-catenin in female rats. This would result in a greater susceptibility to depressive-like behavior in female than in male rats. This effect may be counterbalanced in male rats by increased phosphorylation of GSK3 by the PI3K-Akt pathway leading to no overall effect on β-catenin. This hypothesis would explain, at least in part, the sex difference in the susceptibility to the pro-depressive and pro-anxiogenic effects of D1-D2 heteromer activation in our behavioral models.

The sex differences revealed in the present study, emanating from the higher levels of D1-D2 heteromer, coupled to lower density of D1 receptor in NAc of female rat, and the resulting sex differences in basal and D1-D2 heteromer-induced signaling through BDNF/TrkB and GSK3/β-catenin pathways may be at the origin, or at least represent an essential role, in the usually reported sex difference in female susceptibility to stress, anxiety, and depression. Since the signaling pathways activated by BDNF/TrkB and especially GSK3/β-catenin are ubiquitous, the role of the D1-D2 heteromer in promoting pro-depressive and pro-anxiogenic effects through modulation of these pathways in the basal state and after stress appears more dominant in female rat compared to male. The D1-D2 heteromer may emerge as a novel target in counteracting these effects for the treatment of depression and anxiety in both sexes, but particularly in addressing the increased vulnerabilities to these conditions in the female sex.

## Perspectives and significance section

In this study, we investigated a very specific question related to the fact that depression and anxiety are more common among females than males. Since, it is known that the dopamine D1-D2 heteromer is involved in depression- and anxiety-like behaviors, the possibility that this receptor complex is implicated in the sex-dependent expression of these behavioral alterations has been examined. Moreover, we have also investigated the possible underlying molecular signaling.

This manuscript explored a very relevant issue of strong interest and it could be of great interest for both psychiatrists and for neuroscientists interested in the molecular mechanisms of mood disorders, and notably the related sex differences. We describe a novel potential mechanism through which dopamine may be modulating sex differences in vulnerability to stress- and depression-related behaviors.

Modulation of the dopamine D1-D2 receptor heteromer may represent a novel pharmacological therapeutic target in the treatment of depression and anxiety in both sexes, but particularly in addressing the higher incidence and sensitivity to these conditions in the female sex.

## Supplementary information

**Supplementary Figure 1.** A. Control for co-immuprecipitation and analysis of GAPDH expression. Related to Figure 1.

## Data Availability

All data and material are described succinctly and are available upon request.
