# Supplementary material for: Sex difference in dopamine D1-D2 receptor complex expression and signaling affects depression- and anxiety-like behaviors
Source: Biol Sex Differ. 2020 Feb 22;11:8. doi: 10.1186/s13293-020-00285-9 (PMC7035642; doi:10.1186/s13293-020-00285-9)
Supplement: Supplementary file 1 — Supplementary Figure 1. A. Control for co-immuprecipitation and analysis of GAPDH expression. Related to Figure 1. [file 13293_2020_285_MOESM1_ESM.pdf]

Supplementary Figure 1

A

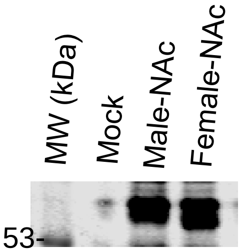

Control for co-immunoprecipitation
Image of D1R co-immunoprecitated with D2R from male and female NAc.
A mock control, IgG with buffer but no NAc proteins, was used as a control. No band corresponding to the D1R was detected, confirming the specificity of the co-immuprecipitation.

B

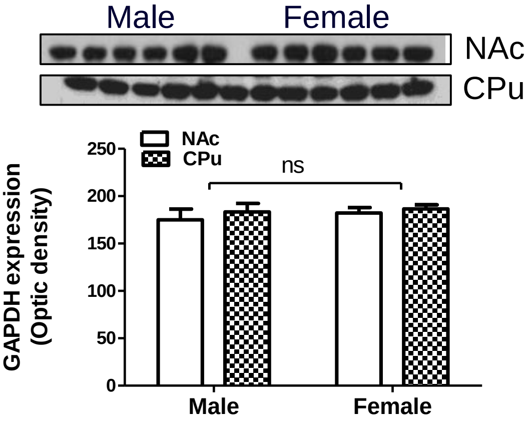

Analysis of GAPDH expression
GAPDH was used as control for all proteins analyzed by western blotting. We tested if this control protein was not sexually dimorphic. Shown is WB images and analysis of GAPDH expression in the NAc and CPu of female and male rats. Statistical analysis (Two-way ANOVA, “sex” and “region”) showed that there is no significant (ns) difference between male and female rats in the expression of GAPDH in the NAc nor in the CPu.
